# Pediatric fractures in northern China: hospital-based epidemiology and high-risk factors from a regional cohort study

**DOI:** 10.3389/fped.2026.1678240

**Published:** 2026-03-11

**Authors:** Fei-hu Li, Bin Dong, Chen-jing Li, Bin-bin Xing

**Affiliations:** Department of Orthopaedics, Yuncheng Central Hospital Affiliated to Shanxi Medical University, Yuncheng, Shanxi Province, China

**Keywords:** epidemiology, hospital-based, pediatric fractures, seasonality, time-of-day

## Abstract

**Objective:**

To identify high-risk factors for pediatric fractures in a hospital-based regional cohort.

**Methods:**

We conducted a retrospective hospital-based cohort study including children ≤15 years treated for fractures at Yuncheng Central Hospital, Shanxi Province, between September 2021 and August 2024. Demographic (age, sex, BMI z-score, residence), injury-related (mechanism, time, season), and fracture characteristics were collected. Case identification and reporting were conducted in accordance with STROBE guidelines. Multivariable logistic regression models were prespecified to estimate adjusted odds ratios (aORs) with 95% confidence intervals (CIs), alongside descriptive statistics.

**Results:**

Among 1,664 cases, males accounted for a significantly higher proportion than females (male:female ratio 1.70:1; 95% CI: 1.54–1.88), with disparity increasing in adolescence. Adolescents (11–15 years) were more frequently hospitalized (31.8% vs. 14.0%, *P* < 0.001). Summer showed the highest case burden, winter the lowest (outpatients 35.4% vs. 17.5%; inpatients 33.5% vs. 15.2%, *P* = 0.12). Daytime injuries predominated in inpatients (53.6% vs. 45.3%, *P* < 0.01). Falls were the leading cause (68.2%), followed by traffic accidents (15.4%), bicycle-related injuries (8.6%), and other mechanisms (7.8%). Radius/ulna and clavicle fractures were commonly managed outpatient, while femoral and distal humeral fractures required hospitalization. Elevated BMI showed no significant association with fracture risk (6.5%).

**Conclusion:**

Pediatric fractures in this regional cohort showed clear differences across sex, age, season, and time of injury. Younger children were mainly affected by fall-related injuries, whereas adolescents experienced more traffic- and activity-related trauma. These patterns suggest that prevention efforts should be tailored to developmental stages, such as improving home safety for preschoolers and reinforcing traffic and sports safety education for older children.

## Introduction

Pediatric fractures remain a frequent reason for clinical visits and are largely the result of accidental injuries. As congenital and disease-related causes of injury have declined with improvements in healthcare and nutrition, unintentional trauma has become a major contributor to morbidity in children (1). Previous epidemiological reports suggest that roughly one quarter of children experience some form of traumatic injury each year, and fractures account for a substantial proportion of these events (2). Seasonal and regional differences in fracture occurrence have been described (3–5), yet how these injuries present across different hospital settings has not been fully characterized.

Climatic conditions and children's daily activity patterns have been linked to variations in fracture incidence in many settings (6–8). For instance, distal radius fractures often peak during winter, although colder regions may paradoxically report fewer cases during the same season compared with temperate areas (9). Despite these observations, detailed cohort studies from single, high-volume regional centers are still limited. Such data are valuable because hospitals in different regions may receive distinct types of cases, influenced by local healthcare access, parental decision-making, and children's living and play environments. These factors can create regional differences not only in overall fracture incidence but also in the types of cases that present to hospitals, which has implications for prevention planning and resource allocation.

This study was carried out in a northern Chinese city (35°N, population approximately five million) with a typical monsoon climate and a single pediatric orthopedic referral center, allowing for comprehensive capture of local fracture cases. Because many continental and temperate regions share similar seasonal patterns, the observations from this setting may also be informative for comparable areas. Over a three-year period, we reviewed pediatric fracture cases to describe demographic patterns, seasonal and temporal trends, injury mechanisms, and fracture characteristics. We also examined how environmental and behavioral factors may contribute to injury risk. Based on prior evidence, we anticipated that sex, age group, season, and time of day would be associated with fracture occurrence. These findings may help guide local prevention efforts and contribute to future regional planning.

## Methods

### Study population

**Figure 1:**
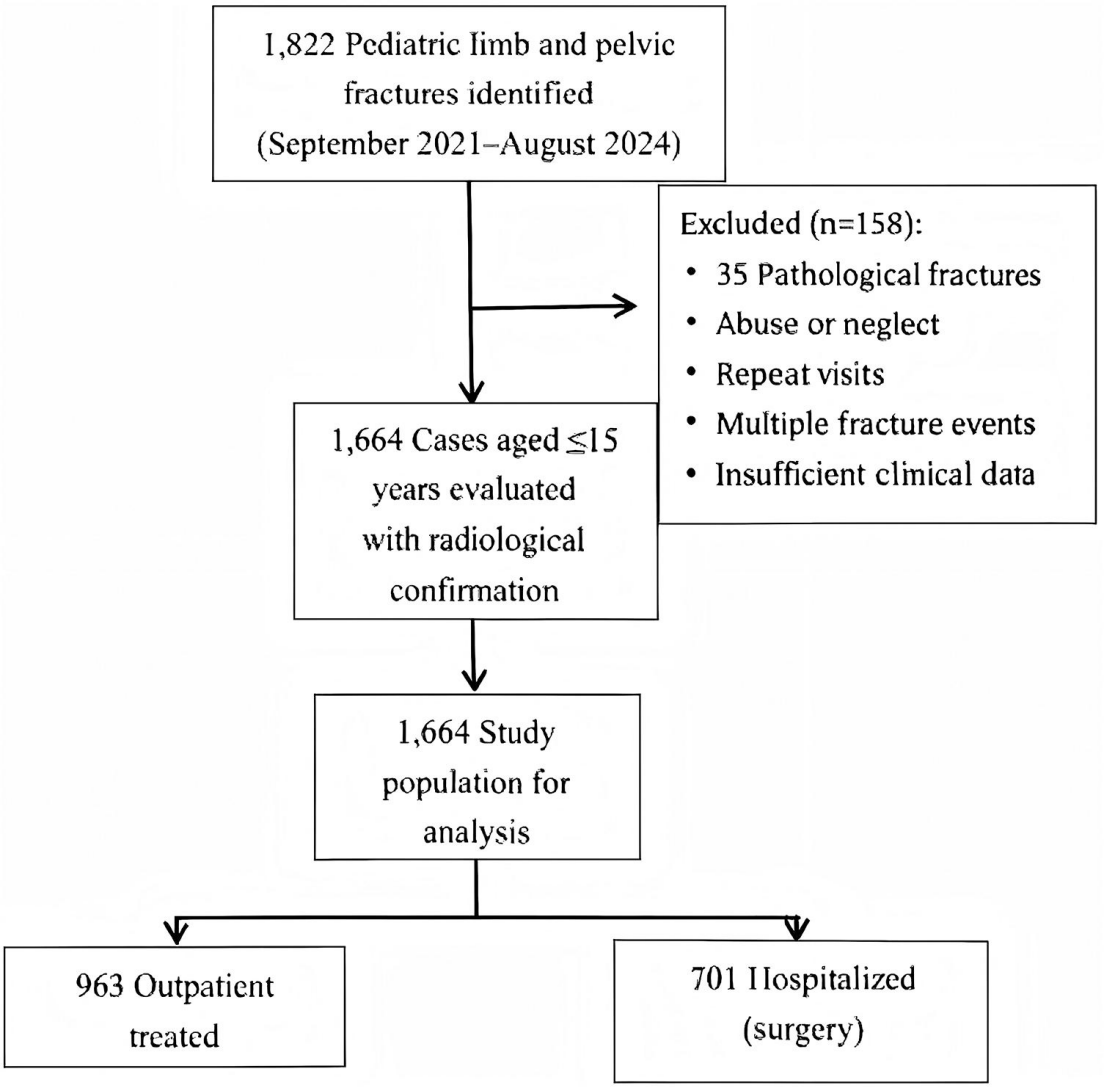
Pediatric fracture cohort selection flowchart.

This study was carried out in Yuncheng, a northern Chinese city with a population of about five million. We included children aged 15 years or younger who were treated for extremity or pelvic fractures at the city's only pediatric orthopedic center between September 2021 and August 2024. All fractures were verified through imaging, including X-ray, CT, or MRI. We excluded cases involving spinal or pathological fractures, suspected abuse, duplicate entries, and repeated fracture events in the same patient. The case selection process is summarized in Figure 1.

### Study design & data collection

We collected information on a range of demographic, clinical, and injury-related variables to describe fracture patterns and explore potential risk factors. Key variables included:

- Demographics: sex, age group (0–5, 6–10, 11–15 years), BMI (WHO z-scores), residence (urban/rural)
- Injury Mechanisms: classified via ICD-10 codes (V01–Y99) (10), including traffic accidents, falls, mechanical injuries, blunt trauma, bicycle-related injuries, and others.
- Temporal Distribution: season (spring, summer, autumn, winter), time of injury (daytime: 07:00–18:00, including morning and afternoon; nighttime: 18:00–07:00)
- Clinical Characteristics: fracture site (e.g., humerus, radius/ulna, femur, tibia/fibula, pelvis) and type (e.g., greenstick, fissure, open/closed).

### Data quality and statistical analysis

Data were extracted from the hospital information system and reviewed independently by two authors. Cases with unclear information were discussed with a senior investigator, and a random 10% sample was checked to maintain data accuracy. Missing values were handled using listwise deletion. High-energy trauma was defined as injuries caused by traffic or bicycle accidents, falls from height, skateboarding, or similar mechanisms. To examine factors associated with hospitalization and high-energy trauma, we used multivariable logistic regression. Adjusted odds ratios (aORs) and 95% confidence intervals (CIs) were calculated with Python (v3.14), and statistical significance was set at *P* < 0.05.

### Ethical approval

The study was approved by the Institutional Review Board (YXLL-YJ2023017). Informed consent was obtained from the parents or legal guardians of all participants.

## Results

### General characteristics

Between September 2021 and August 2024, our hospital treated 1,664 children aged 15 years or younger for limb or pelvic fractures. Among them, 701 patients (42.1%) were admitted for surgical management, while the rest received outpatient care. Key demographic and clinical characteristics—including age, sex, BMI, residence, season, and treatment setting—are presented in Table 1.

**Table 1:** Baseline characteristics of outpatient and inpatient groups.

Characteristic	Outpatient (*n* = 963)	Inpatient (*n* = 701)	*P* value
Age group (0–5/6–10/11–15 yrs)	32.1/ 45.3/ 22.6%	31.1/37.4/ 31.5%	<0.001
Sex (Male %)	62.1%	64.1%	0.42 (NS)
BMI ≥25 (%)	NA	6.5%	NA
Residence (Urban %)	NA	46.5%	NA
Time of occurrence (Day vs. Night)	45.3% vs. 54.7%	53.6% vs. 46.4%	<0.01
Season (Summer vs. Winter)	35.4% vs. 17.5%	33.5% vs. 15.2%	0.12 (NS)
Injury mechanism (Falls/Traffic/Bicycle/Other)	NA	68.2%/15.4%/8.6%/7.8%	NA

Values are presented as percentages unless otherwise indicated. NA, not available; data only collected for inpatients. NS, not significant.

### Age and gender distribution

**Figure 2:**
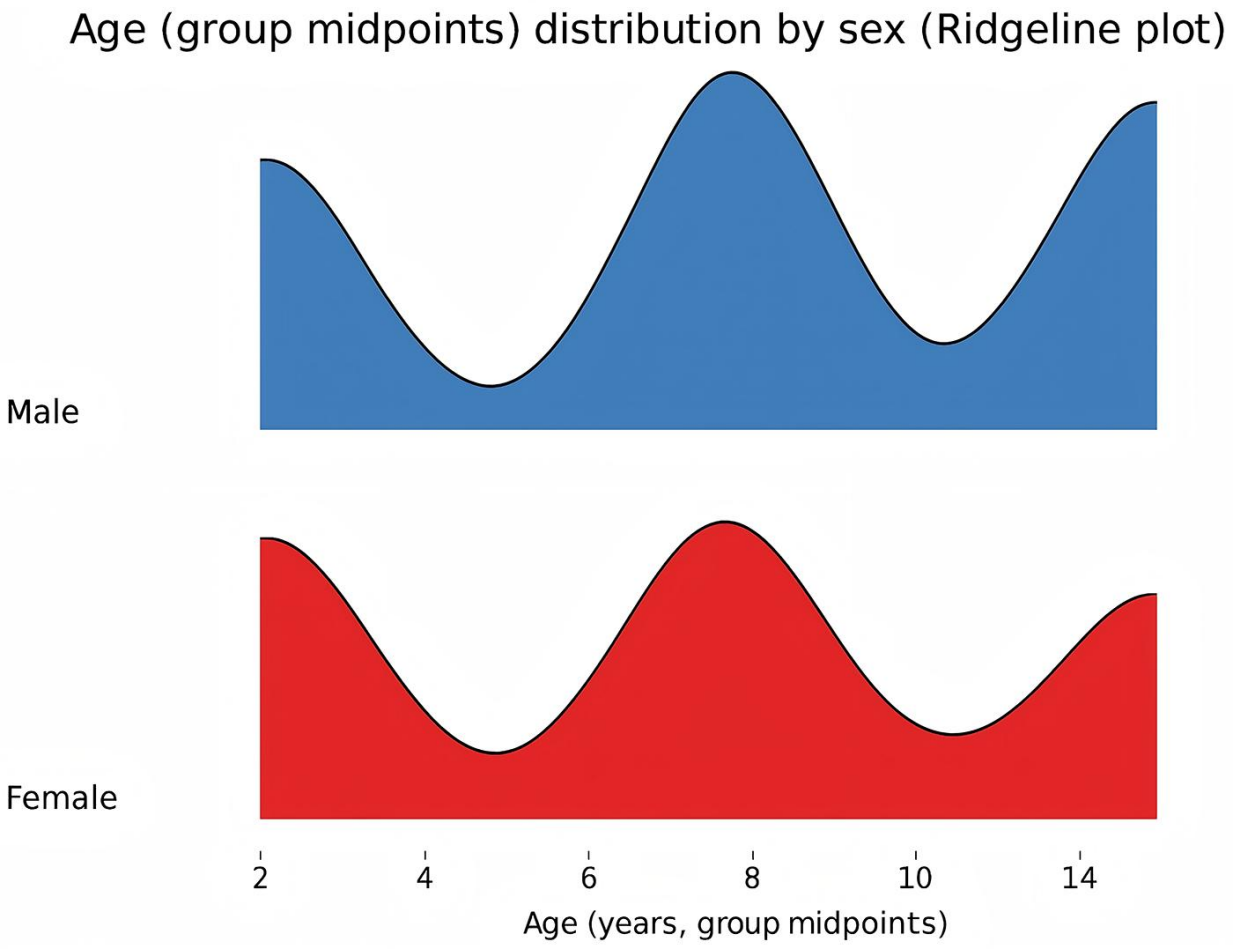
Age and gender distribution chart.

A total of 1,047 boys and 617 girls were included, giving a male-to-female ratio of 1.70 (95% CI: 1.54–1.88). The proportion of boys rose with age (*χ*^2^ = 47.59, *P* < 0.001) and reached 70.8% in the 11–15-year group (95% CI: 66.6–75.1; Figure 2). Among hospitalized patients, 322 (46.5%) lived in urban areas and 378 (54.0%) in rural areas. The relative proportion of urban to rural cases was slightly lower (RR = 0.85, 95% CI: 0.73–0.99), suggesting broadly similar distributions.

### Temporal and seasonal distribution

**Figure 3:**
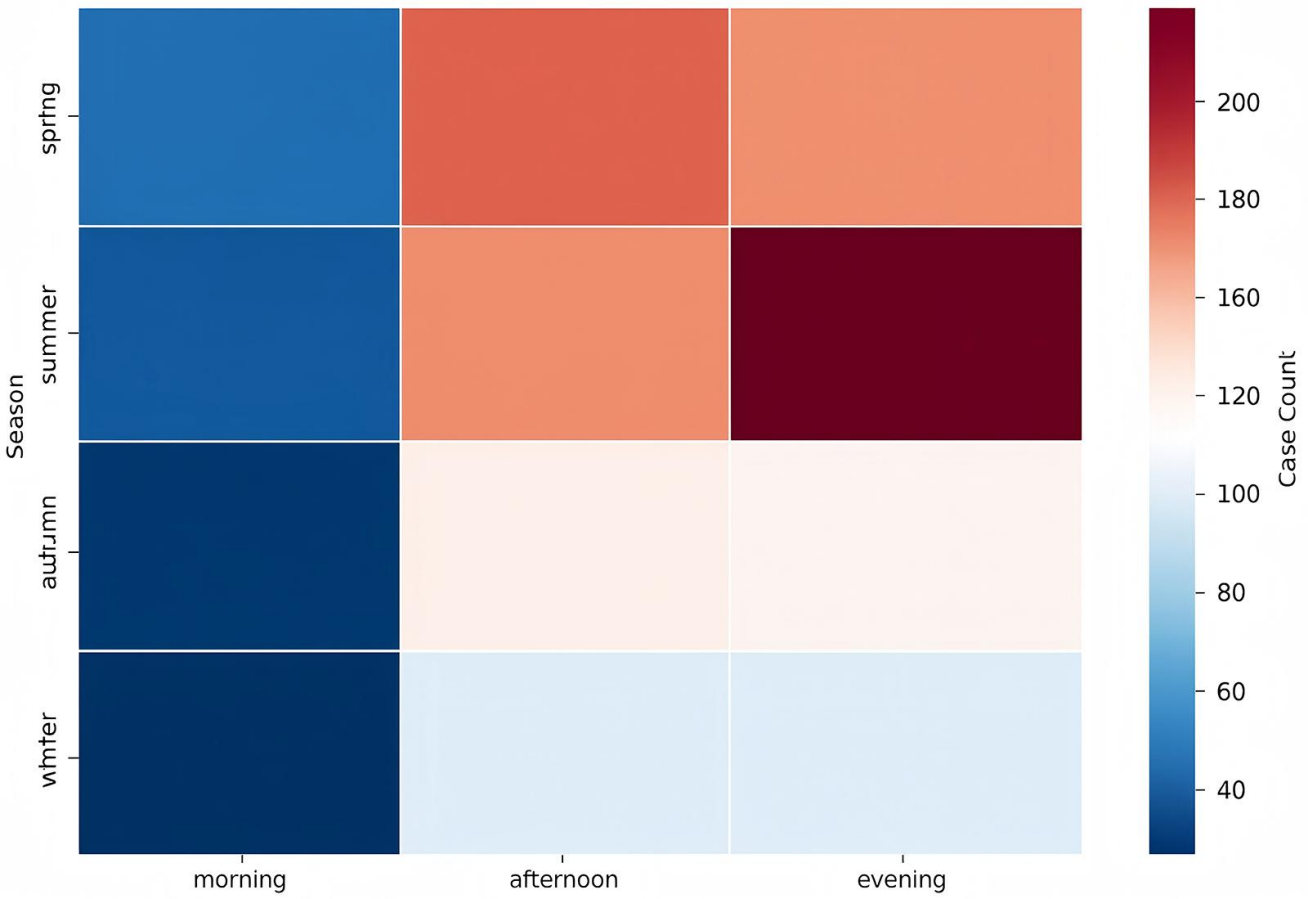
Heatmap of fracture cases by season and time of day.

Seasonal variation was evident, with the highest number of fractures occurring in summer (34.5%) and the lowest in winter (16.5%; *χ*^2^ = 109.0, *P* < 0.001). Compared with summer, winter showed a markedly lower risk (RR = 0.48, 95% CI: 0.41–0.55). Injuries also varied by time of day: 57.9% occurred during daytime hours and 42.1% at night (RR = 1.37, 95% CI: 1.24–1.51; Figure 3).

### Injury mechanisms and fracture patterns

**Figure 4:**
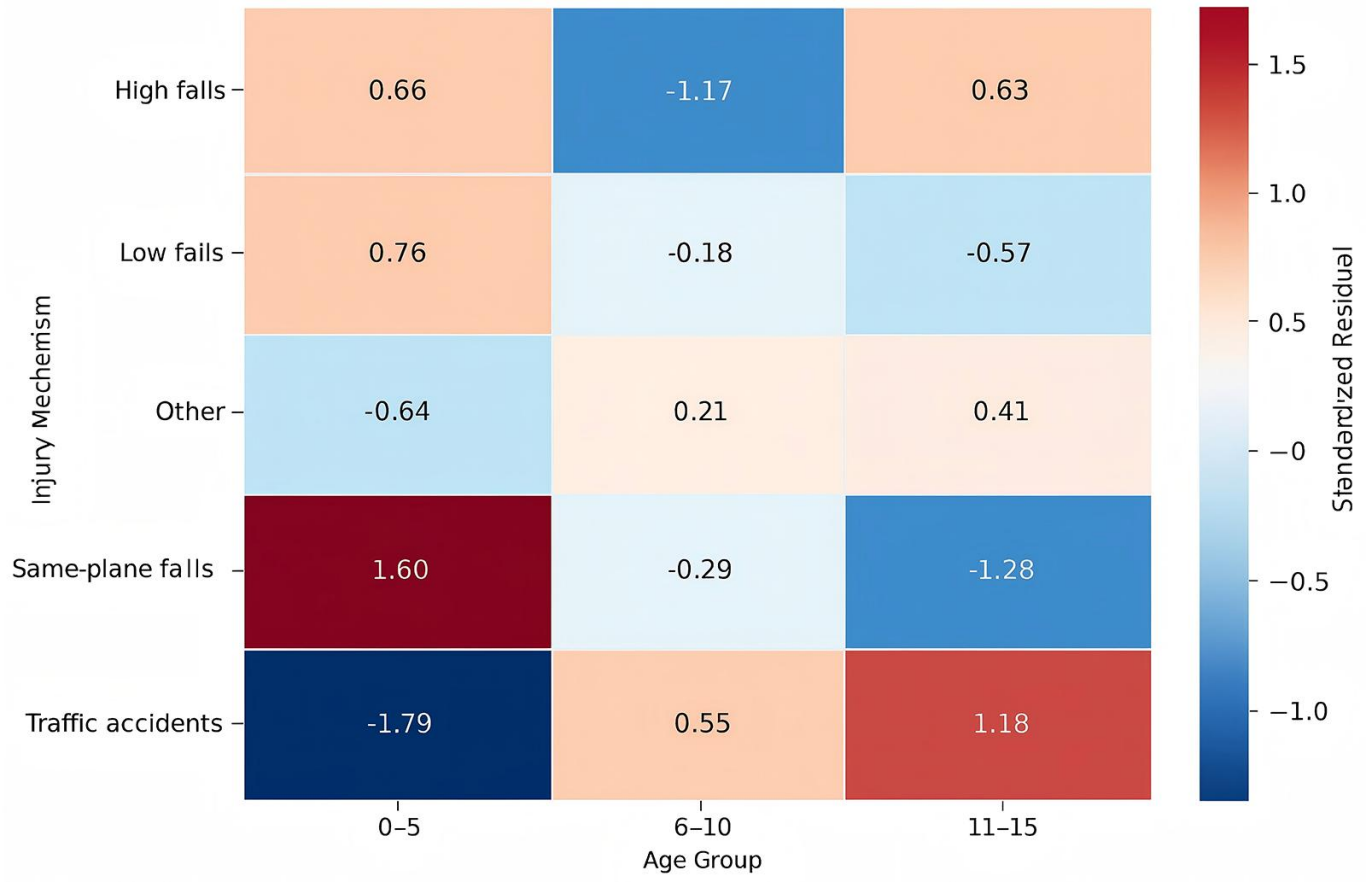
Standardized residuals of injury mechanisms by age group.

**Figure 5:**
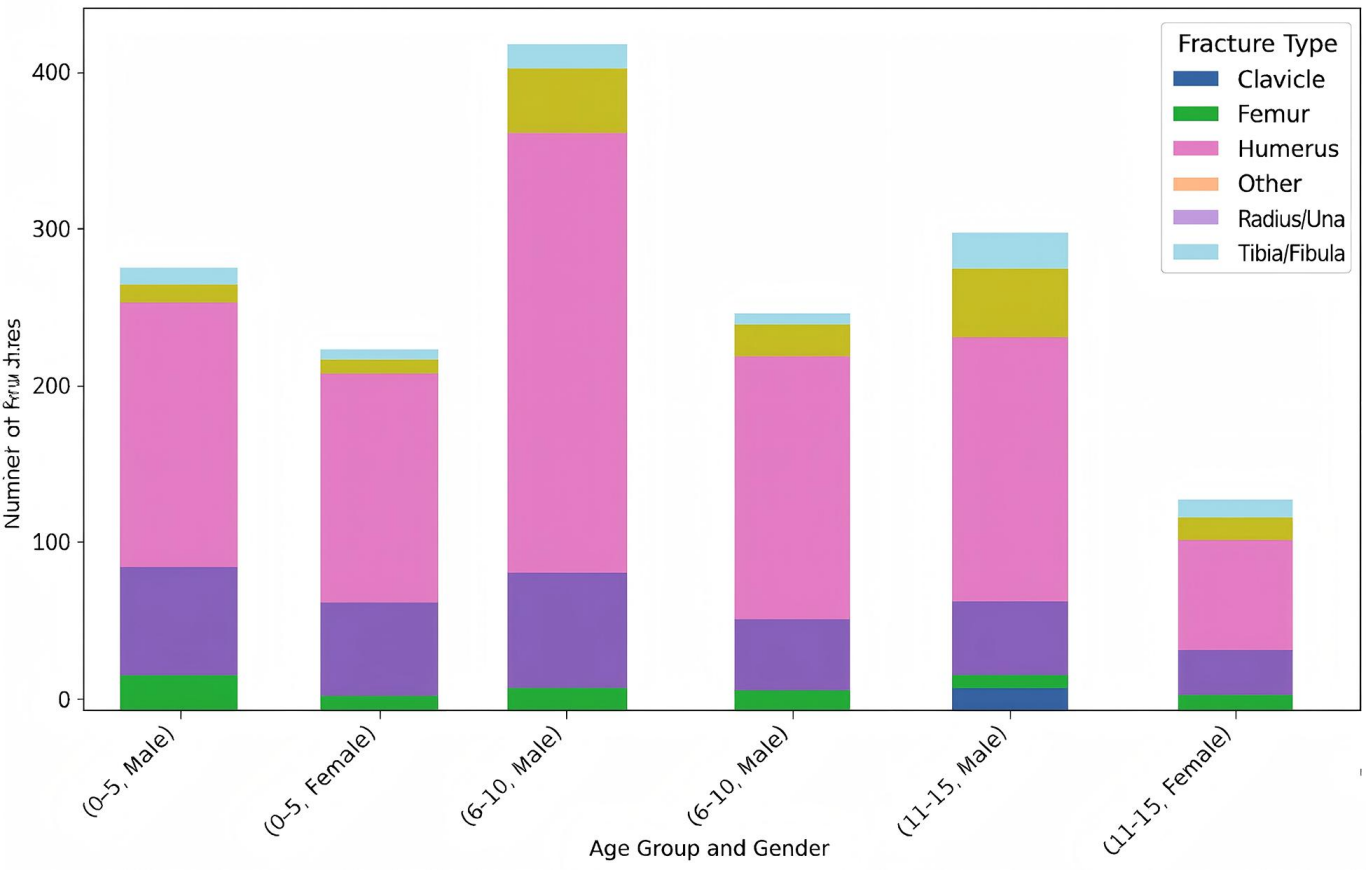
Distribution of fracture types by age group and gender.

Ground-level falls were the main cause of injury in younger children, whereas adolescents experienced relatively more traffic- and bicycle- related trauma. Fracture patterns also differed by age: supracondylar humerus fractures were most common in preschoolers, clavicle and distal radius fractures were frequent in school- aged children, and forearm fractures were more typical among adolescents (Figures 4, 5).

### Multivariable analysis of high-energy trauma

**Figure 6:**
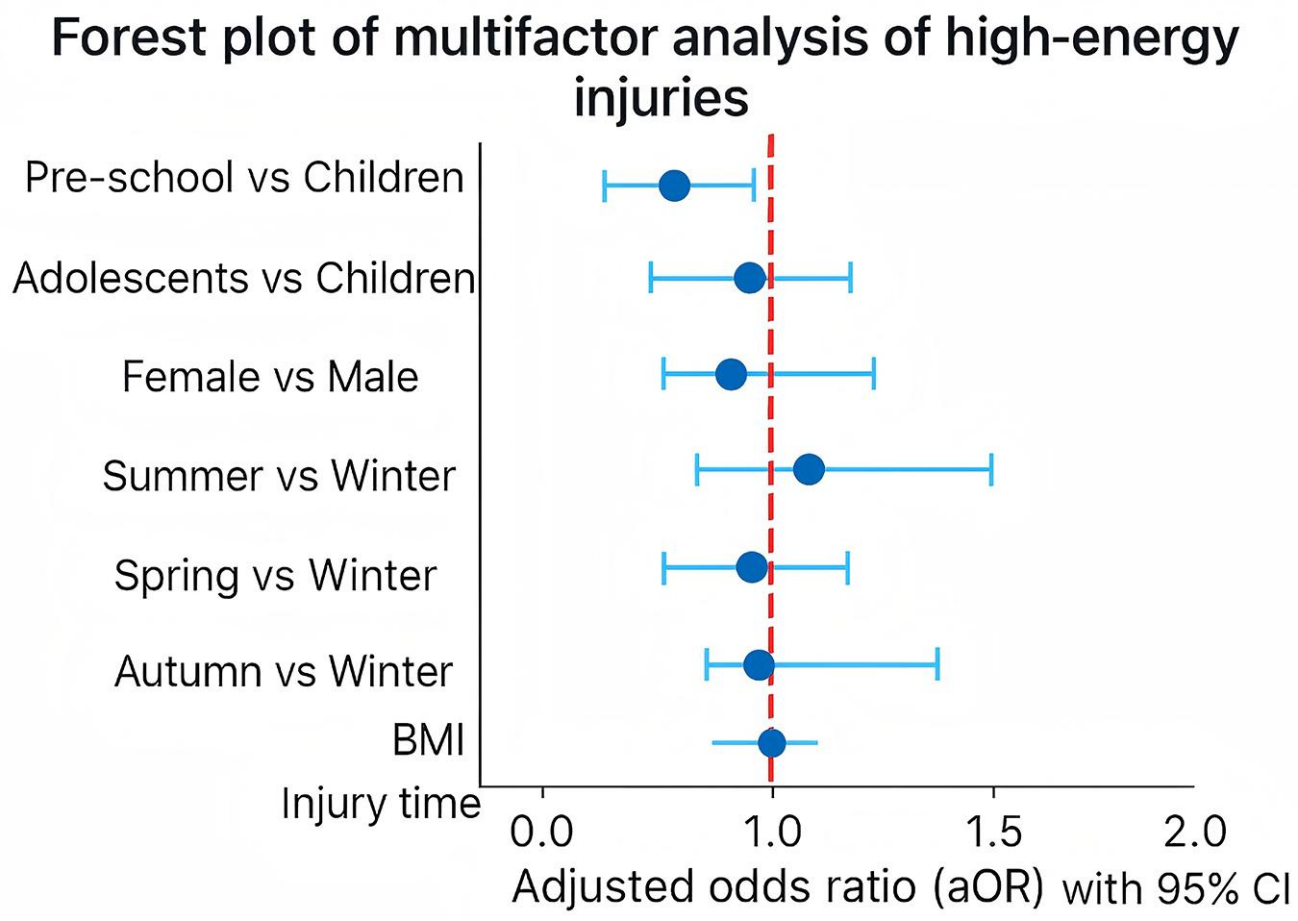
Forest plot of multivariable logistic regression analysis for risk factors of high-energy trauma among hospitalized pediatric fracture patients.

High-energy trauma included injuries caused by traffic or bicycle accidents, falls from height, skateboarding, and similar mechanisms. In multivariable analysis, adolescents had higher odds of sustaining high-energy trauma (aOR = 1.79, 95% CI: 1.06–3.02), as did children injured at night (aOR = 1.45, 95% CI: 1.01–2.08). Compared with winter, both summer (aOR = 0.44, 95% CI: 0.26–0.75) and autumn (aOR = 0.51, 95% CI: 0.27–0.97) were associated with lower risk. Preschool children also showed reduced odds (aOR = 0.48, 95% CI: 0.30–0.78). No significant associations were observed for sex, elevated BMI, or spring season. Results are shown in Figure 6.

## Discussion

In this cohort, notable differences in fracture patterns were observed across age and sex. Boys accounted for a larger proportion of cases, a trend also described in previous studies and often attributed to higher activity levels and greater exposure to risk during adolescence (11). The mechanisms of injury varied by developmental stage: younger children were mainly injured by ground-level falls, school-aged children experienced more object-related trauma, and adolescents were more frequently involved in traffic- or bicycle-related incidents. These observations point to the need for age-appropriate prevention efforts, such as closer supervision for preschoolers and strengthened traffic safety education for older children. Similar age-stratified patterns have been reported in other regions, including findings from Shenzhen, where fall-related injuries predominated in younger children and traffic- or sports-related trauma increased with age (12).

Clear seasonal differences were observed, with more fractures occurring during summer and fewer in winter. These fluctuations likely reflect changes in outdoor activity associated with weather conditions. Injuries were also more common in the afternoon and evening. Although this pattern may simply mirror higher activity levels later in the day, we cannot rule out the possibility that some families delay seeking care; our dataset did not include time-to-presentation information to explore this further (13). Similar seasonal and diurnal trends have been described in studies from Switzerland, Malawi, and other regions, suggesting that environmental exposure and daily activity routines play a consistent role in pediatric fracture risk (14–16).

The distribution of injury mechanisms in our cohort differed somewhat from reports in other regions. For example, the proportion of fall-related fractures was higher than that reported by Mansoor K and colleagues (51% vs. 44.2%), whereas the proportion of traffic-related injuries was similar (22.9% vs. 26.7%) (17). These variations may be influenced by differences in local environments, community safety practices, and healthcare-seeking behaviors, and they underscore the importance of considering regional context when comparing epidemiological data.

Upper-limb fractures were the most frequent injuries in our cohort, with supracondylar humeral fractures particularly common in younger children. Femoral fractures, in contrast, were more likely to require hospitalization. These patterns highlight the importance of appropriate triage and timely surgical evaluation for high-energy trauma (18, 19). Prevention efforts may also benefit from being tailored to developmental stages. For younger children, closer supervision and safer home environments could help reduce fall-related injuries. School- aged children may benefit from safety education focused on playground and sports activities, while adolescents could be encouraged to use protective equipment such as bicycle helmets to reduce the risk of traffic- and cycling-related fractures.

## Conclusion

Pediatric fractures in this regional cohort varied by sex, age, season, and injury mechanism, with boys and adolescents showing the highest overall risk. Younger children were mainly affected by fall-related injuries, while adolescents experienced more traffic- and sports-related trauma. Upper-limb fractures were the most common overall, and femoral fractures were more likely to require hospitalization. These patterns suggest that prevention and clinical management may benefit from being tailored to different age groups. Although this study reflects the experience of a single center, the findings offer useful epidemiological information that may help guide future multicenter work and support the development of locally appropriate injury-prevention strategies.

## Limitations

This study has several limitations that should be taken into account when interpreting the results. First, it was conducted at a single tertiary care center, which may limit the generalizability of the findings to other settings. Second, referral bias may exist, as more severe cases are likely to seek hospital care, potentially inflating the proportion of high-energy injuries and surgical admissions. Third, the study lacked denominator data on the underlying population distribution by age group, preventing calculation of true incidence rates and restricting comparisons across age strata. These factors highlight that our results should be interpreted cautiously and may not be universally applicable. Future multicenter studies incorporating population-based data are needed to validate and extend these findings.

## Data Availability

The original contributions presented in the study are included in the article/Supplementary Material, further inquiries can be directed to the corresponding author.
