# Supplementary material for: Pediatric fractures in northern China: hospital-based epidemiology and high-risk factors from a regional cohort study
Source: Front Pediatr. 2026 Mar 11;14:1678240. doi: 10.3389/fped.2026.1678240 (PMC13013465; doi:10.3389/fped.2026.1678240)
Supplement: Supplementary file 1 [file Datasheet1.pdf]

STROBE Statement—checklist of items that should be included in reports of observational studies

	Item No	Recommendation
**Title and abstract**	1	(a) Yes — The title and abstract indicate the study design ("retrospective hospital-based cohort study").
		(*b*) Yes — The abstract provides an informative and balanced summary of objectives, methods, results, and conclusions.
**Introduction**		
Background/rationale	2	Yes — Background and rationale are explained in the Introduction.
Objectives	3	Yes — Specific objectives are stated, including prespecified hypotheses.
**Methods**		
Study design	4	Yes — Study design is presented early in the Methods section.
Setting	5	Yes — Setting, location (Yuncheng Central Hospital, Shanxi Province), and study period (Sep 2021–Aug 2024) are described.
Participants	6	Yes — Eligibility criteria (≤15 years, fracture cases) and selection methods are reported.
		(b) Not applicable — No matched design.
Variables	7	Yes — Outcomes, exposures, predictors, and confounders are clearly defined.
Data sources/ measurement	8*	Yes — Data sources and measurement methods are described.
Bias	9	Yes — Potential referral bias is acknowledged.
Study size	10	Yes — Study size is explained (all eligible cases during study period).
Quantitative variables	11	Yes — Quantitative variables (e.g., BMI z-score) are defined and categorized.
Statistical methods	12	(a) Yes — Statistical methods, including logistic regression and confounder adjustment, are described.
		(b) Yes — Subgroup analyses (urban/rural, BMI categories) are reported.
		(c) Yes — Missing data handling is described.
		(d) Not applicable — No loss to follow-up in retrospective design
		(e) Yes — Sensitivity analyses (excluding re-visits and multiple fractures) are reported.

Continued on next page

**Results**		
Participants	13*	(a) Yes — Numbers of eligible, included, and analyzed cases are reported (n=1664).
		(b) Yes — Reasons for exclusion are described.
		(c)Yes — Participant flow is summarized.
Descriptive data	14*	(a) Yes — Characteristics of participants (age, sex, BMI, residence) are reported.
		(b) Yes — Missing data are indicated.
		(c) Not applicable — No follow-up time in retrospective design.
Outcome data	15*	Yes — Outcome data (fracture types, mechanisms, hospitalization) are reported.
Main results	16	(*a*) Yes — Both unadjusted and adjusted estimates with 95% CI are reported.
		(*b*) Yes — Category boundaries (e.g., age groups, BMI categories) are defined.
		(*c*) Not applicable — No absolute risk translation.
Other analyses	17	Yes — Subgroup and sensitivity analyses are reported.
**Discussion**		
Key results	18	Yes — Key results are summarized with reference to objectives.
Limitations	19	Yes — Limitations (single-center, referral bias, lack of denominator data) are discussed.
Interpretation	20	Yes — Interpretation is cautious, considering objectives, limitations, and prior studies.
Generalisability	21	Yes — Generalisability is discussed.
**Other information**		
Funding	22	Yes — Funding source and role of funders are stated (no external funding).

*Give information separately for cases and controls in case-control studies and, if applicable, for exposed and unexposed groups in cohort and cross-sectional studies.

Note: An Explanation and Elaboration article discusses each checklist item and gives methodological background and published examples of transparent reporting. The STROBE checklist is best used in conjunction with this article (freely available on the Web sites of PLoS Medicine at http://www.plosmedicine.org/, Annals of Internal Medicine at http://www.annals.org/, and Epidemiology at http://www.epidem.com/). Information on the STROBE Initiative is available at www.strobe-statement.org.
